# Brachial thromboembolectomy and retrograde innominate artery stenting in acute limb ischemia

**DOI:** 10.1016/j.jvscit.2024.101675

**Published:** 2024-11-08

**Authors:** Rachel Bernardo, Hamda Almaazmi, Shawn Sarin, Salim Lala

**Affiliations:** a The George Washington University School of Medicine and Health Sciences, Washington, DC; b Department of Surgery, George Washington University Hospital, Washington, DC; c Department of Radiology, George Washington University Hospital, Washington, DC

**Keywords:** Acute limb ischemia, Innominate artery thrombus, Retrograde stenting, Thromboembolectomy, Vascular emergency

## Abstract

Acute limb ischemia is a critical vascular emergency often resulting from embolic sources, requiring prompt intervention to prevent significant morbidity and mortality. This paper presents a case of a 74-year-old female with acute limb ischemia due to a thromboembolus in the distal brachial artery and a nonocclusive mobile thrombus in the innominate artery. The patient underwent urgent brachial artery thromboembolectomy and subsequent retrograde innominate artery stenting via right open transcarotid approach. The retrograde approach was chosen to minimize stroke risk associated with embolization. The successful resolution of the arterial thrombus and restoration of arterial patency underscore the importance of individualized management strategies in complex vascular emergencies.

Acute limb ischemia (ALI) due to embolic sources is a critical vascular emergency with an incidence of about 1.5 cases per 10,000 person-years.^1^ This condition is marked by a sudden blockage of blood flow to a limb, leading to significant morbidity and mortality if not treated promptly.^2^ The therapeutic strategy for ALI depends on multiple factors, including the location, Rutherford class at presentation, duration of ischemia, comorbidities, and therapy-related risks and outcomes.^3^ Endovascular techniques, such as percutaneous catheter-directed thrombolysis,^4^ percutaneous thromboaspiration, and percutaneous mechanical thrombectomy,^5^ aim to restore blood flow with minimal invasiveness, whereas open surgery, including thrombectomy and bypass, is preferred for time-urgent cases or when endovascular methods are contraindicated.^3^ Both approaches have shown similar outcomes in terms of mortality and limb salvage, but the choice of technique depends on individual patient characteristics and specific clinical scenarios.^6^

Potential embolic causes of ALI include cardiac embolization, aortic embolization, thrombosed grafts, hypercoagulable states, paradoxical venous-to-arterial embolism, and iatrogenic complications related to endovascular procedures.^7^ A thrombus in the innominate artery presents unique therapeutic challenges due to its contribution to both cerebral and upper extremity circulation and the risks of distal embolization via the carotid and subclavian arteries.^8^ Treatment options for innominate artery occlusion include various antegrade or retrograde endovascular catheter-based techniques, often in combination with surgical exposure of the common carotid artery.^9^ We present a case involving a 74-year-old female with a thromboembolic occlusion in the distal brachial artery and a nonocclusive thrombus in the innominate artery. She was managed with an urgent brachial artery thromboembolectomy followed by retrograde innominate artery stenting. The patient provided consent for the report of her case and associated images.

## Case report

A 74-year-old female with hypertension, hyperlipidemia, and hypothyroidism presented to the emergency department with acute right hand pain and numbness for 2 hours. She denied similar previous episodes or a history of blood clots. On arrival, she was afebrile and hemodynamically stable. Her right hand was paler and cooler than the left, with a monophasic right radial signal and absent right ulnar and right brachial pulses. Sensation was decreased, but motor function remained intact. Laboratory results showed a white blood cell count of 13,000/mm³, with other parameters normal. A computed tomography angiography of the right upper extremity revealed a filling defect in the distal brachial artery at the elbow with distal reconstitution without stenosis or atherosclerotic disease, suggestive of occlusion.

The patient was taken to the operating room for a right upper extremity thromboembolectomy. A longitudinal incision exposed the pulseless right brachial artery. Proximal control was obtained, and the patient was heparinized. A longitudinal arteriotomy was made, and a #3 Fogarty catheter was passed proximally to remove the thrombus until good forward bleeding was achieved. The #3 and #2 Fogarty catheters were passed distally into the radial and ulnar arteries, respectively, with significant thrombus return and backbleeding noted. The arteriotomy was repaired with a bovine pericardial patch. After releasing the clamps, hemostasis was confirmed, and a palpable radial pulse was noted at the right wrist. Doppler confirmed triphasic signals in the radial and ulnar arteries and palmar arch.

**Fig 1:**
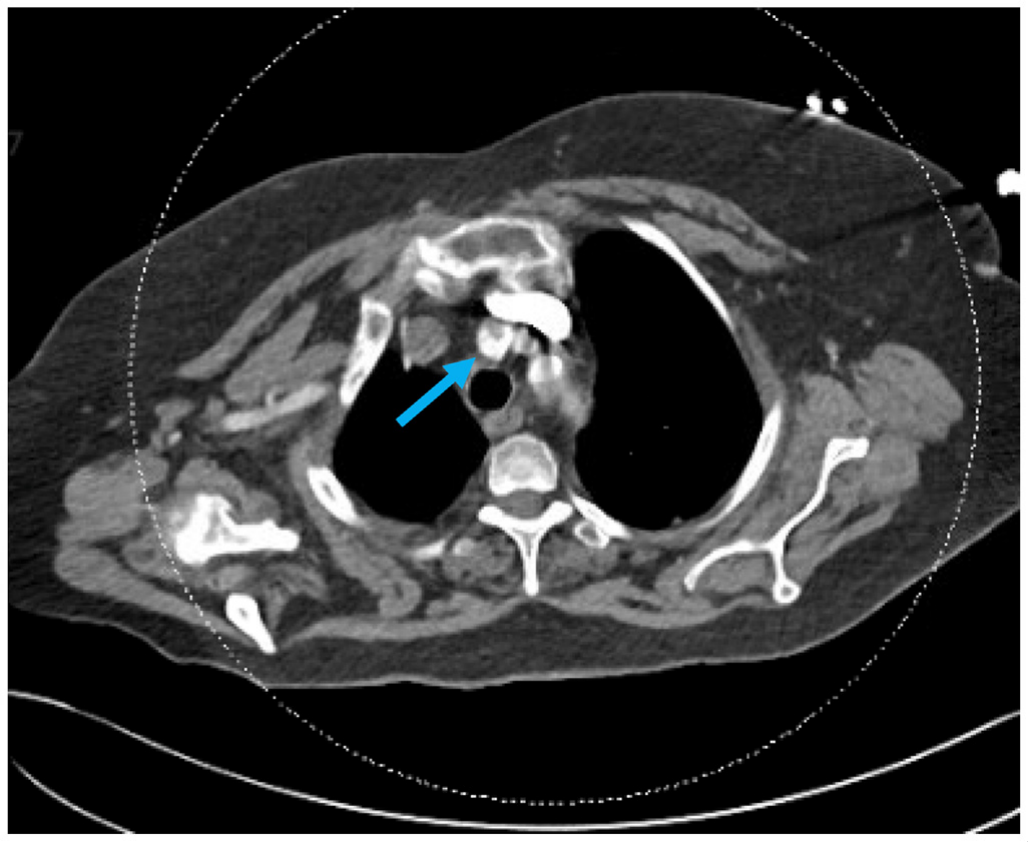
Computed tomography angiography (*CTA*) with contrast showing nonocclusive thrombus in the innominate artery from its origin to just proximal to its bifurcation (*blue arrow*).

Postoperative evaluation included a computed tomography angiography of the chest to rule out atrial thrombus, which revealed a nonocclusive thrombus in the right innominate artery from its origin to just proximal to its bifurcation (Fig 1). No evidence of left atrial or ventricular thrombus was found. A transthoracic echo was also performed that demonstrated normal left and right ventricular function with no significant valvular dysfunction. The etiology of the thrombus remains uncertain, but it was most likely due to the rupture of an atherosclerotic plaque.

**Fig 2:**
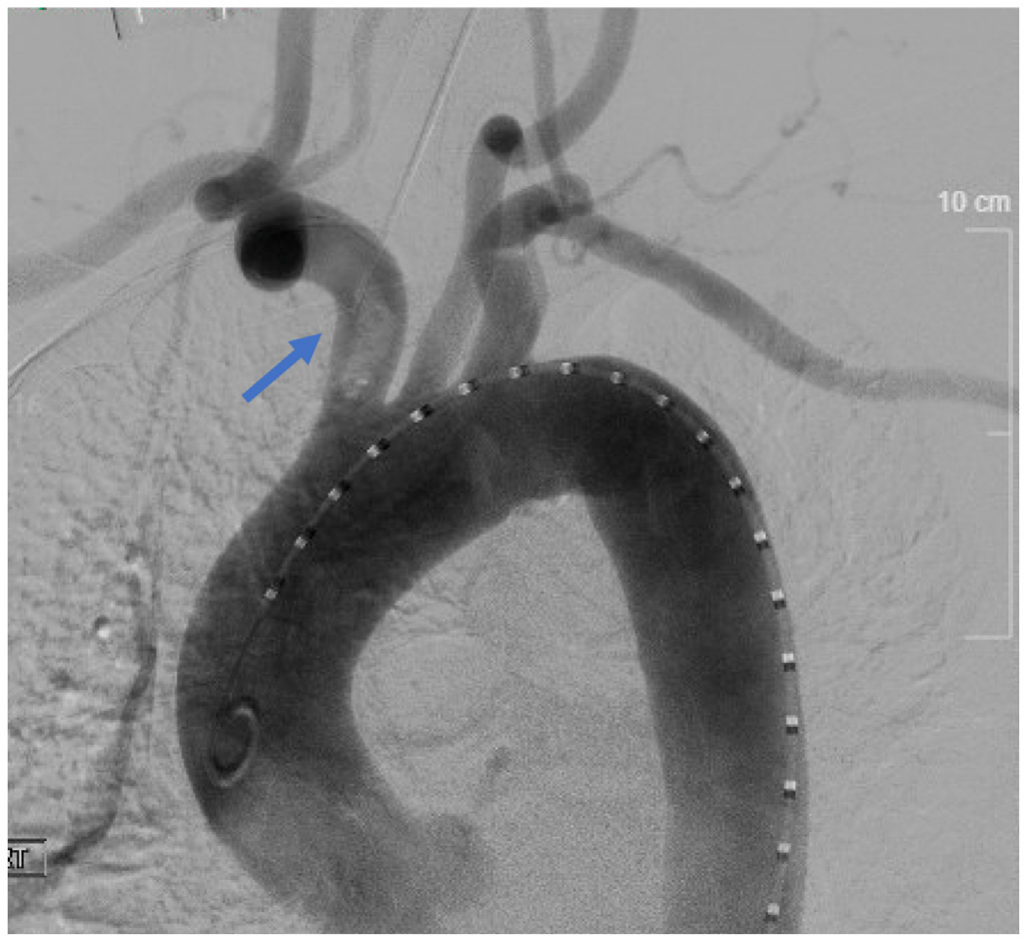
Arch aortogram revealing widely patent aortic arch with patent right innominate artery and demonstration of a nonocclusive mobile thrombus within the right innominate artery (*blue arrow*).

Due to the high risk of embolization to the cerebral circulation or recurrence of limb ischemia, the decision was made to perform right innominate artery stenting. Under general anesthesia, a longitudinal incision was made in the right neck. The right common carotid artery was dissected out with proximal and distal control obtained. Ultrasound-guided access to the right common femoral artery was obtained and upsized to a 6 French sheath. A pigtail catheter was introduced into the ascending aorta, and an arch aortogram revealed the mobile nonocclusive thrombus in the innominate artery (Fig 2).

**Fig 3:**
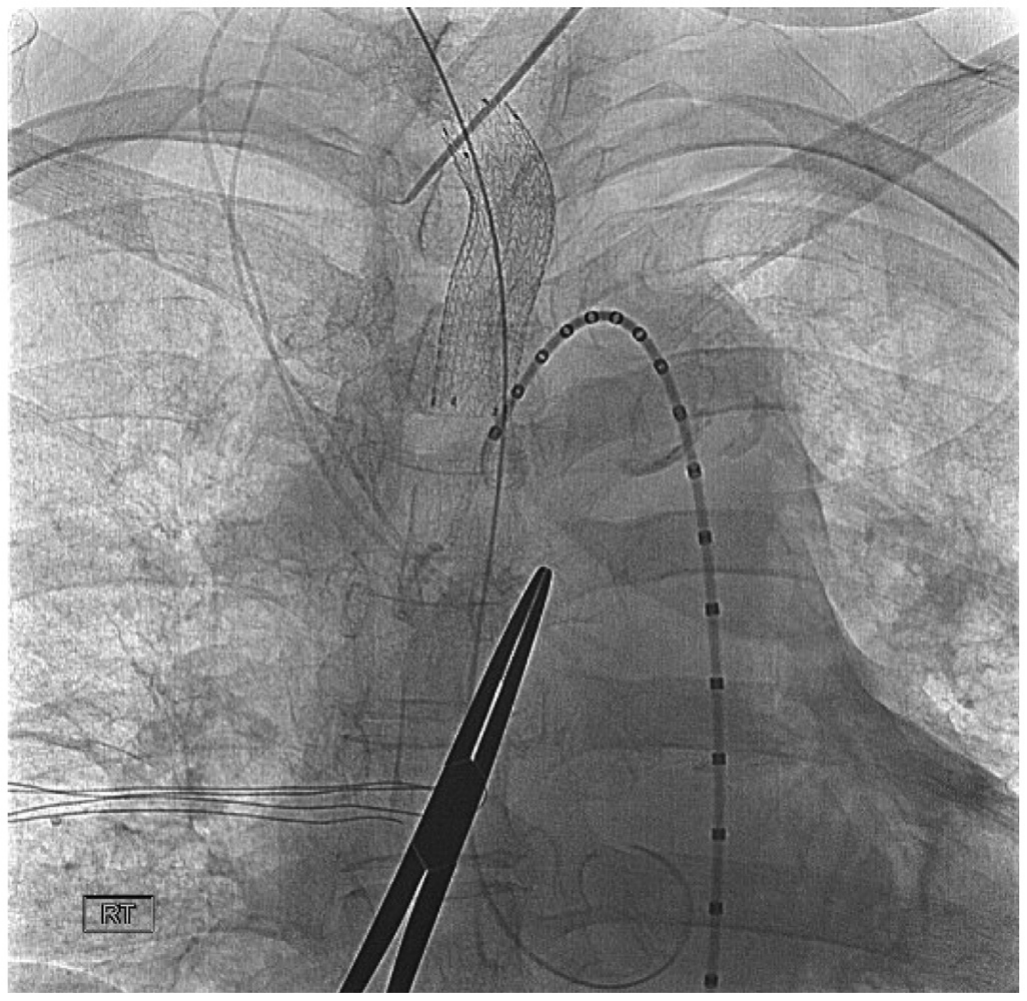
GORE VIABAHN self-expanding endoprosthesis (13 mm × 5 cm) (W. L. Gore & Associates, Inc) stent deployed in the right innominate artery with no visualization of the mobile thrombus.

To prevent distal embolization, the distal common carotid artery was clamped, maintaining a mean arterial pressure over 90 mmHg. Micropuncture entry into the right common carotid artery was obtained in a retrograde fashion and upsized to a 5 French sheath over a 0.035 Glidewire. The Glidewire was passed beyond the innominate artery thrombus into the ascending aorta, and the sheath was upsized to a 10 French sheath. A 13 mm × 5 cm GORE VIABAHN self-expanding endoprosthesis (W. L. Gore & Associates, Inc) was deployed into the right innominate artery. A completion arteriogram showed a widely patent stent with no evidence of free-floating thrombus (Fig 3). The 10 French sheath was removed from the right neck, and proximal control was obtained with another vascular clamp. A transverse arteriotomy was made in the right common carotid artery, and the proximal clamp was released to flush out any potential thrombus from the carotid circulation. The proximal common carotid artery was reclamped, and the transverse arteriotomy was repaired. After appropriate flushing maneuvers, the clamps were released, and hemostasis was achieved. Right radial artery access was then obtained, and angiograms were performed for the right brachial artery, carotid, cerebral, and vertebral arteries that were patent and without evidence of embolization. At the end of the procedure, the intraoperative heparin was reversed with protamine sulfate. A postoperative ultrasound confirmed antegrade flow in the right vertebral artery.

The patient tolerated the procedure well and was monitored in the intensive care unit with regular neurovascular checks. She was initially started on a heparin drip with dual antiplatelet therapy. On post-op day 2, the heparin was transitioned to apixiban. She exhibited stable vital signs and no signs of recurrent ischemia or embolization. She also had a hematology workup and was notably negative for JAK2 and antiphospholipid antibody syndrome. Follow-up imaging with computed tomography scan confirmed widely patent innominate stent with no evidence of recurrent thrombus or embolic complications. The patient was discharged home in stable condition with appropriate follow-up with vascular surgery two weeks post-discharge.

## Discussion

The innominate artery’s involvement in both cerebral and upper extremity circulation presents a unique therapeutic challenge, with significant risks of distal embolization via the carotid and subclavian arteries.^10^^,^^11^ Endovascular and surgical treatments yield similar postoperative stroke rates of 2.1% and 2.5%, respectively.^12^ Retrograde stenting through surgical access to the right common carotid artery provides a controlled environment to reduce the potential for embolic events to the cerebral circulation.

Although antegrade innominate stenting is a viable option, it carries a higher risk of cerebral embolization due to the direct passage of catheters and devices through the aortic arch and carotid artery, which can dislodge plaques or thrombi, leading to a higher risk of stroke.^13^ The retrograde approach, involving accessing the lesion from the carotid artery, allows for better control and minimizes the risk of embolization to the brain by allowing for arterial clamping^9^^,^^14^ and flushing^12^ during the procedure.

During the stenting procedure, embolic protection for the vertebral and right subclavian arteries was not feasible due to anatomical constraints, specifically the acute angulation of the vertebral artery from the subclavian artery. The risk of embolization to the vertebral artery was considered low relative to the risk of embolization to the cerebral circulation via the right common carotid artery, which would have more devastating consequences. Our focus was to prioritize protection of the brain, as upper extremity embolization is more manageable either endovascularly or surgically.

Retrograde innominate artery stenting was successfully performed in our patient to manage a nonocclusive thrombus, effectively preventing stroke and recurrent limb ischemia. This approach, favored over antegrade stenting due to the lower stroke risk, underscores the importance of tailored interventions in complex vascular cases for optimal patient outcomes. The decision to use retrograde stenting was based on its high technical success rate, lower complication rates, and the specific anatomical considerations of the innominate artery. This case highlights the effectiveness of retrograde stenting as a preferred method in managing innominate artery thrombi.

## Conclusion

Retrograde innominate artery stenting was successfully performed to manage a mobile nonocclusive thrombus, effectively preventing stroke and recurrent limb ischemia. This approach, favored over antegrade stenting due to lower stroke risk, highlights the importance of tailored interventions in complex vascular cases for optimal patient outcomes.

## Disclosures

None.
